# Addition of CORES to the I-PASS Handoff: A Resident-led Quality Improvement Study

**DOI:** 10.1097/pq9.0000000000000251

**Published:** 2020-01-22

**Authors:** Lauren M. Tufts, Christopher L. Damron, Susan L. Flesher

**Affiliations:** From the Marshall Health Department of Pediatrics, Marshall University, Huntington, W.Va.

## Abstract

Quality patient handoff is vital in patient care and attainable with structured handoff systems, such as the I-PASS mnemonic. This paper describes a continuous quality improvement study occurring after the implementation of the I-PASS handoff bundle. Our objectives were to (1) determine compliance with the inclusion of I-PASS elements during handoff and (2) determine whether the addition of CORES, an electronic tool that generates a patient list designed for use with I-PASS, would improve compliance and sustainability.

**Methods:**

We developed an aim statement: 90% of handoffs would include all 6 I-PASS elements within 6 months of the addition of CORES. Two plan–do–study–act (PDSA) cycles were conducted. In PDSA 1, we implemented CORES. In PDSA 2, we reeducated residents on I-PASS elements and the importance of a quality handoff. We used a checklist to evaluate the inclusion of I-PASS elements. Following PDSA 2, we administered a survey regarding CORES to involved residents.

**Results:**

During PDSA 1, illness severity, diagnosis, patient summary, contingency planning, action list, and receiver synthesis were present in 13%, 62%, 52%, 87%, 42%, and 25% of handoffs, respectively. Overall compliance was 47%. During PDSA 2, illness severity remained stable at 13% whereas the remainder increased to 84%, 82%, 93%, 91%, and 37%. Overall compliance increased to 67%. Following PDSA 2, 100% of survey respondents reported improved handoff with CORES.

**Conclusions:**

In this study, we show that neither implementation of CORES nor resident reeducation resulted in the return to high postintervention compliance observed after implementation of the I-PASS handoff bundle.

## INTRODUCTION

The importance of quality patient handoff in decreasing sentinel events and improving patient care^1^ continues to gain popularity in the literature. The I-PASS handoff bundle is at the forefront of this transition. This handoff bundle is based on the evidence-based I-PASS mnemonic, which represents the essential elements of a successful handoff process: I, illness severity; P, patient summary; A, action items; S, situational awareness and contingency planning; S, synthesis by receiver.^2^

After developing the I-PASS mnemonic in 2012, Starmer et al applied the project to 9 residency programs in the United States and Canada with a reduction in the overall medical error rate by 23% and a decrease in preventable adverse events by 30%.^2^ The I-PASS mnemonic continues to prove its efficacy in producing a positive transformational change in handoff structure across several academic medical centers.^3,4^

Despite this positive change, limitations to I-PASS exist, the most evident of which is the maintenance of bundle compliance. Several studies documented potential barriers to achieving sustained practice change,^5,6^ including the absence of a structured electronic handoff tool to be used alongside the I-PASS mnemonic.^7,8^ In a 2016 study by Clarke et al, implementation of a structured electronic tool resulted in improved trainee handoff compliance from 73% to 96% and decreased communication error 50%, with improved efficiency and workflow.^9^

One such structured electronic tool is the CORES handoff program by TransformativeMed Inc. (Seattle).^10^ This program integrates into the Cerner electronic medical record (North Kansas City, MO) and formulates patient lists centered around the I-PASS mnemonic. Specifically, CORES organizes each patient’s medical information into the 6 key elements of I-PASS. The program also auto-populates up-to-date information onto the patient list, including new laboratory and diagnostic imaging studies, negating the need for our residents to update these portions of the patient list manually. The situational awareness portion of the patient list requires manual updates by our residents but will auto-populate any previously input information that is not deleted.

Before this study, a resident-driven project was performed to determine whether a multidisciplinary quality improvement process could improve the inclusion of I-PASS elements in the resident handoff. After completion of 3 plan–do–study–act (PDSA) cycles, the aim to include all 6 I-PASS elements in at least 90% of handoffs was accomplished.^11^ However, this improvement was not sustained. Compliance with all 6 elements had deteriorated during the next 18 months.

This study is a continuous quality improvement project occurring after the initial successful implementation of the I-PASS handoff bundle. It is unique in that it continues to be resident driven. The primary question of our study was to determine compliance with the inclusion of I-PASS elements during handoff and whether the addition of CORES would improve compliance and sustainability. We created an aim statement for the process measure: 90% of handoffs would include all 6 I-PASS elements within 6 months of the addition of CORES.

## METHODS

### Setting

The setting is a 25-bed pediatric unit within a children’s hospital affiliated with an academic institution. The pediatric medical team typically consists of 1 attending physician, 2–3 senior residents, and 3–4 interns. One intern resident works at night and is directly supervised by 1 senior resident, who is also responsible for covering the pediatric intensive care unit. Interns with senior resident supervision lead the verbal handoff between day and night shifts.

### Intervention Planning

A senior resident recognized the positive transformational change after the initial implementation of the I-PASS mnemonic and also recognized the potential for poor sustained compliance with the current written handoff process. Written handoff consisted of a patient list in the form of an excel spreadsheet. Residents updated the spreadsheet manually with new results and changes in treatment. This senior resident saw that resident satisfaction and workflow could be improved with the implementation and integration of CORES into the handoff. The senior resident was compelled to lead a quality improvement team to achieve these goals. In developing this project, the resident used the Kurt Lewin change theory, a 3-step model of “unfreeze, change, freeze,”^12^ as a guide for successful project execution and sustainment of positive outcomes.

Our team consisted of 1 attending pediatric hospitalist, 1 senior resident, and 1 medical student. We planned to evaluate the process measure of the inclusion of individual I-PASS elements in the handoff between residents during each PDSA cycle. We used this process measure to evaluate the outcome measure of compliance with the inclusion of all 6 key elements during handoff. This project was a quality improvement project and not human subject research. Therefore, the institutional review board did not require review and approval.

### Implementation of the I-PASS Handoff Bundle

Implementation of the I-PASS handoff bundle is outlined in the previously published article “Quality Improvement Regarding Handoff” by Studeny et al.^11^ We use the results of that study as the targeted goal for the present study, whereby illness severity, diagnosis, patient summary, contingency planning, action list, and receiver synthesis were present in 97%, 100%, 100%, 100%, 100%, and 97% of handoffs, respectively.^11^

### PDSA Cycle 1 Intervention

Residents expressed dissatisfaction with the patient list on surveys administered during the implementation of the I-PASS handoff bundle.^11^ Therefore, we implemented the CORES handoff program. The information technology staff integrated this program into the Cerner electronic medical record. The program generated a patient list in which each of the 6 key elements was organized and presented for each patient to guide residents through the I-PASS handoff process.

Residents were involved in the creation of the patient list, including what information should auto-populate. Residents were given no formal training on the CORES handoff program upon completion, as all participated in the development of the program and were therefore familiar with it. An example of the CORES patient list is shown in Table 1. This first PDSA cycle occurred 18 months following the implementation of the original I-PASS handoff bundle.

**Table 1.**
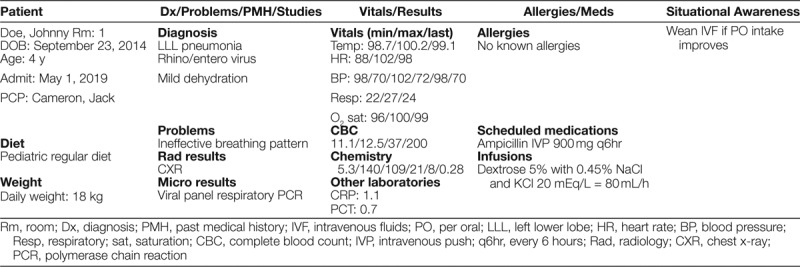
Sample CORES Patient List

### PDSA Cycle 2 Intervention

This intervention involved the reeducation of the residents. This education was less involved than the education courses completed during the implementation of the I-PASS handoff bundle. Here, all residents were given a 1-hour lecture by the chief resident. During this lecture, the chief resident reminded the residents of the 6 key elements of a quality handoff and the importance of accurate communication during patient care. This PDSA cycle occurred 6 months following PDSA 1.

### Postintervention Survey

Following PDSA 2, our attending pediatric hospitalist and medical student developed and administered a survey to all involved residents for the attainment of qualitative data. We surveyed 24 residents anonymously on the CORES handoff tool and the perceived difficulties in maintained compliance in the inclusion of I-PASS elements during patient handoff. Written comments were encouraged. This survey is shown in Table 2.

**Table 2.**
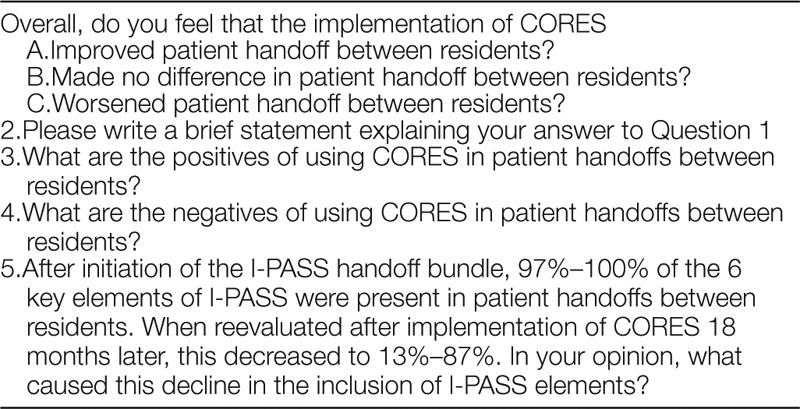
Resident Survey

### Data Collection and Analysis

We collected the process data that included the individual 6 key elements of the I-PASS mnemonic using a checklist. On this checklist, the observer documented whether or not each patient handoff included the elements of illness severity, diagnosis, patient summary, contingency planning, action list, and receiver synthesis. We calculated the averaged percent inclusion of all 6 key elements to determine the outcome measure of overall compliance. The total time for handoff was documented and evaluated as a balancing measure. Verbal handoff occurred between recurring day and night shift teams throughout the week and between random resident coverage on weekends. Both morning and evening handoffs were observed on weekdays and weekends. On average, residents included 15 patients during each handoff.

A medical student member of our quality improvement team completed the data collection. The student was trained in I-PASS along with residents during the implementation of the handoff bundle and acted as an independent observer during handoff. The team participating in handoff was not involved in data collection. The medical student acted as the sole observer in each PDSA cycle, including those throughout the implementation of the I-PASS handoff bundle, for consistency in data collection. The checklist used for data collection is shown in Table 3. We analyzed data for statistical significance using Fisher’s exact test.

**Table 3.**
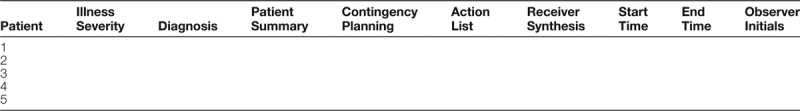
Checklist Used During Handoff to Evaluate Inclusion of I-PASS Elements

### Timing of Study

We conducted this study during late winter and early spring months to ensure that every intern resident had experience giving verbal handoff before data collection.

## RESULTS

**Fig. 1.**
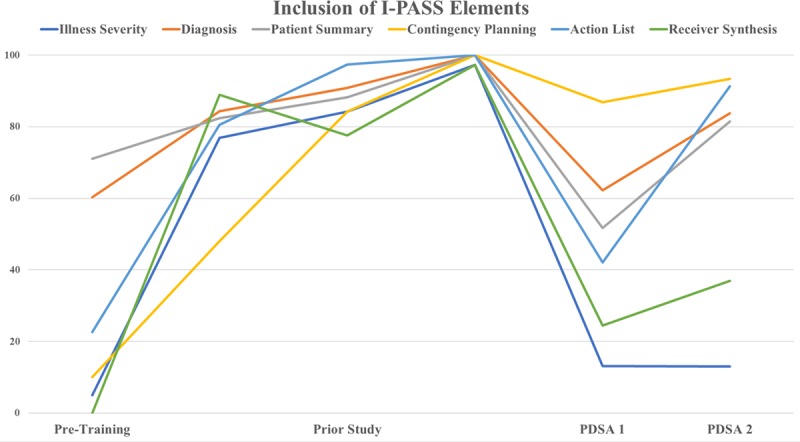
Percent inclusion of I-PASS elements during each PDSA cycle.

During the study period, the medical student observed verbal handoffs on a total of 428 individual patients for the inclusion of the 6 elements, 222 after PDSA 1, and 206 after PDSA 2. During PDSA 1, element compliance of illness severity, diagnosis, patient summary, contingency planning, action list, and receiver synthesis was 13%, 62%, 52%, 87%, 42%, and 25%, respectively. Overall compliance was 47%. During PDSA 2, documentation of illness severity remained stable at 13%, whereas the other elements of diagnosis, patient summary, contingency planning, action list, and receiver synthesis increased to 84%, 82%, 93%, 91%, and 37%, respectively. Overall compliance increased to 67%. Handoff time per patient decreased from an average of 1.85 minutes in PDSA 1 to 1.01 minutes in PDSA 2. Full compliance data after each PDSA cycle is shown in Figure 1.

In the postintervention survey, 100% of surveyed residents reported that the implementation of CORES improved patient handoff between residents. Residents believed this improvement occurred because of the auto-population of patient information by the CORES handoff tool. Specifically, 1 resident stated, “CORES improved handoff and decreased error on documenting medications, labs, etc. because this information is auto-populated. It has streamlined the checkout process and cut down on time spent updating the list, which gives more time to education and patient care.” Many positives of CORES were identified, including handoff becoming more “efficient and streamlined” and that it “served as a reminder to talk about contingency plans and rank sickness of patients.” However, respondents also identified negatives. Residents reported that CORES made the handoff feel “redundant when known facts of long-term patient’s history were repeated” and reported information was “too much for straightforward patients, making it feel like an easy patient took too long to go through.” Another identified negative was that “residents would forget to manually add patients to the CORES list if they were admitted from another service, and the night resident would not receive handoff on these patients.” When asked to comment on the decline of inclusion of 6 key elements of I-PASS over time, residents attributed this decline to “infrequent reeducation,” “poor dissemination to younger classes,” and that I-PASS was “never really ingrained into our culture, making it easy to go back to our old handoff style.”

## DISCUSSION

With hopes of improving resident satisfaction and patient care, this project began with resident-driven integration of the CORES handoff program via a quality improvement project. In the interim 18-month time-lapse between the implementation of the I-PASS handoff bundle and integration of the CORES handoff tool, we report a significant decrease in the inclusion of the 6 key elements during handoff. The inclusion of the 6 key elements improved after completion of resident reeducation 6 months later. However, neither implementation of CORES nor resident reeducation was followed by a return to the high compliance observed after implementation of the original I-PASS handoff bundle.

We note that compliance in every key element improved after resident reeducation except patient illness severity. We believe this may be due to excluding all phrases other than “stable, watcher, or unstable” when collecting data for that key element. For example, if a resident described a patient as “doing well” during his or her handoff, we did not equate that to “stable” nor document that the resident included patient severity in the handoff. This difference in resident style and verbiage may have been a barrier to improvement in the inclusion of that key element.

Our results reflect that we continue to face the challenge of culture change after the implementation of the I-PASS handoff bundle. We cannot obtain culture change until we “freeze” our changes, as outlined in the Kurt Lewin change theory. We believe our interventions were not followed by a return to previously high postimplementation levels because our lack of reeducation never allowed for “freezing” after changes were made. This deficiency was made evident by resident responses in the administered survey. For example, 1 respondent stated, “We do not reevaluate I-PASS often enough to make it our culture. We discussed the process at the beginning of the residency, but the process was discussed minimally afterward. Perhaps readdressing I-PASS once every quarter would be beneficial to helping the residents implement I-PASS more often and efficiently. It can be discussed at our monthly resident forum or between lectures. Also, making sure the senior residents are trained effectively so that the process will be passed down to the interns may be beneficial.”

Furthermore, our results indicate that although CORES improved workflow, it did not in itself produce improved handoffs. This result may be due to the overreliance on the computer program by our residents. Our residents may not focus on ensuring all key elements are included in their verbal handoff if each key element is present on the patient list. However, it is also possible that compliance would have been lower in the 18-month time-lapse if we had not implemented CORES. We did not evaluate these data, which is a limitation of the study.

We conclude that education is the key to facing the challenge of culture change, which in turn will improve compliance with and sustainability of the I-PASS handoff. Therefore, we plan to reeducate our senior residents in the late spring or early summer months before incoming intern residents begin. This education will remind senior residents of the importance of quality handoff and prompt their use of I-PASS, so that quality verbal handoff may be modeled for the incoming intern residents. Second, we plan to educate incoming intern residents on the entire I-PASS handoff bundle during their orientation. By doing these educational interventions with both senior and intern residents, we hope I-PASS will become the culture of patient handoff and improve sustained compliance at our institution.

In this study, we measured the inclusion of the key elements but did not evaluate the accuracy of spoken content and patient-related details during handoff. Therefore, our future goals are not only to maintain compliance with the I-PASS handoff but also to evaluate and ensure accurate handoffs. Once we achieve both compliance and accuracy, we plan to evaluate for relationships with adverse events and medication errors to determine any difference after implementation of the I-PASS handoff. Lessons learned here will guide our attainment of these future goals and our global aim to improve patient safety.

## Disclosure:

The authors have no financial interest to declare in relation to the content of this article.
